# The CPNE Family and Their Role in Cancers

**DOI:** 10.3389/fgene.2021.689097

**Published:** 2021-07-23

**Authors:** Haicheng Tang, Pei Pang, Zhu Qin, Zhangyan Zhao, Qingguo Wu, Shu Song, Feng Li

**Affiliations:** ^1^Department of Respiratory and Critical Care Medicine, Shanghai Public Health Clinical Center, Fudan University, Shanghai, China; ^2^Department of Pathology, The First Affiliated Hospital of Soochow University, Suzhou, China; ^3^Department of Pathology, Shanghai Public Health Clinical Center, Fudan University, Shanghai, China

**Keywords:** copines, cancer, proliferation, metastasis, signaling pathway

## Abstract

Lung cancer is the leading cause of cancer-related deaths worldwide. Despite significant advances in cancer research and treatment, the overall prognosis of lung cancer patients remains poor. Therefore, the identification for novel therapeutic targets is critical for the diagnosis and treatment of lung cancer. CPNEs (copines) are a family of membrane-bound proteins that are highly conserved, soluble, ubiquitous, calcium dependent in a variety of eukaryotes. Emerging evidences have also indicated CPNE family members are involved in cancer development and progression as well. However, the expression patterns and clinical roles in cancer have not yet been well understood. In this review, we summarize recent advances concerning CPNE family members and provide insights into new potential mechanism involved in cancer development.

## Introduction

Recently, based on its increased incidence and mortality, lung cancer has been listed as the leading cause of cancer-related deaths worldwide among males and females (Chen et al., 2016; Siegel et al., 2017). The application of traditional chemotherapeutic drugs can achieve response in some patients; however, these agents lack tissue specificity. Normal cells, especially bone marrow hematopoietic cells and epithelial cells in various organs, such as cells in the gastrointestinal tract, can also be killed by chemotherapeutic drugs (Perez-Herrero and Fernandez-Medarde, 2015; Somaiah et al., 2018). Therefore, traditional treatment methods are characterized by bottlenecks, limited means, and poor efficacy. And recurrence and metastases often develop in patients undergoing traditional treatments. In recent years, the concept of precision medicine has emerged, and corresponding treatments have been developed based on tumor tissue-specific gene changes. For example, genes, such as epidermal growth factor receptor (EGFR), anaplastic lymphoma kinase (ALK), and transmembrane receptor tyrosine kinase (ROS1), are detected in patient tissue specimens (Zhuang et al., 2019), and Corresponding first-, second- or even third-generation drugs have been developed to target these genes. In addition, RET rearrangement, NTRK fusion, BRAF mutation, MET14 mutation and HER2 mutation are also present in lung cancer (Staley et al., 1991; Farago and Azzoli, 2017; Liu et al., 2018a). At present, targeted therapy is mainly applied in patients with advanced non-small cell lung cancer. Despite remarkable breakthroughs in diagnosis and treatment, patient survival rates and treatment results have not changed significantly (Mayekar and Bivona, 2017; Ruiz-Cordero and Devine, 2020). The 5-year survival rate remains only 15%, so it is important to develop new treatment strategies (Mulshine and Sullivan, 2005). Numerous potential mechanisms are involved in invasion and metastasis in non-small cell lung cancer, including tumor microenvironment (Wu et al., 2021). With the emergence of protein chips and gene chips, numerous lung cancer-related genes have been discovered, and it is important to identify specific and sensitive targets. Researchers are working to develop specific targeted drugs and to identify effective biomarkers for the prevention and diagnosis of non-small cell lung cancer. Several proteins have been identified as biomarkers and drug targets, but their exact role remains controversial. Therefore, there is a need to identify reliable biomarkers that can be used as new therapeutic targets for the effective treatment in non-small cell lung cancer patients.

**Figure 1:**
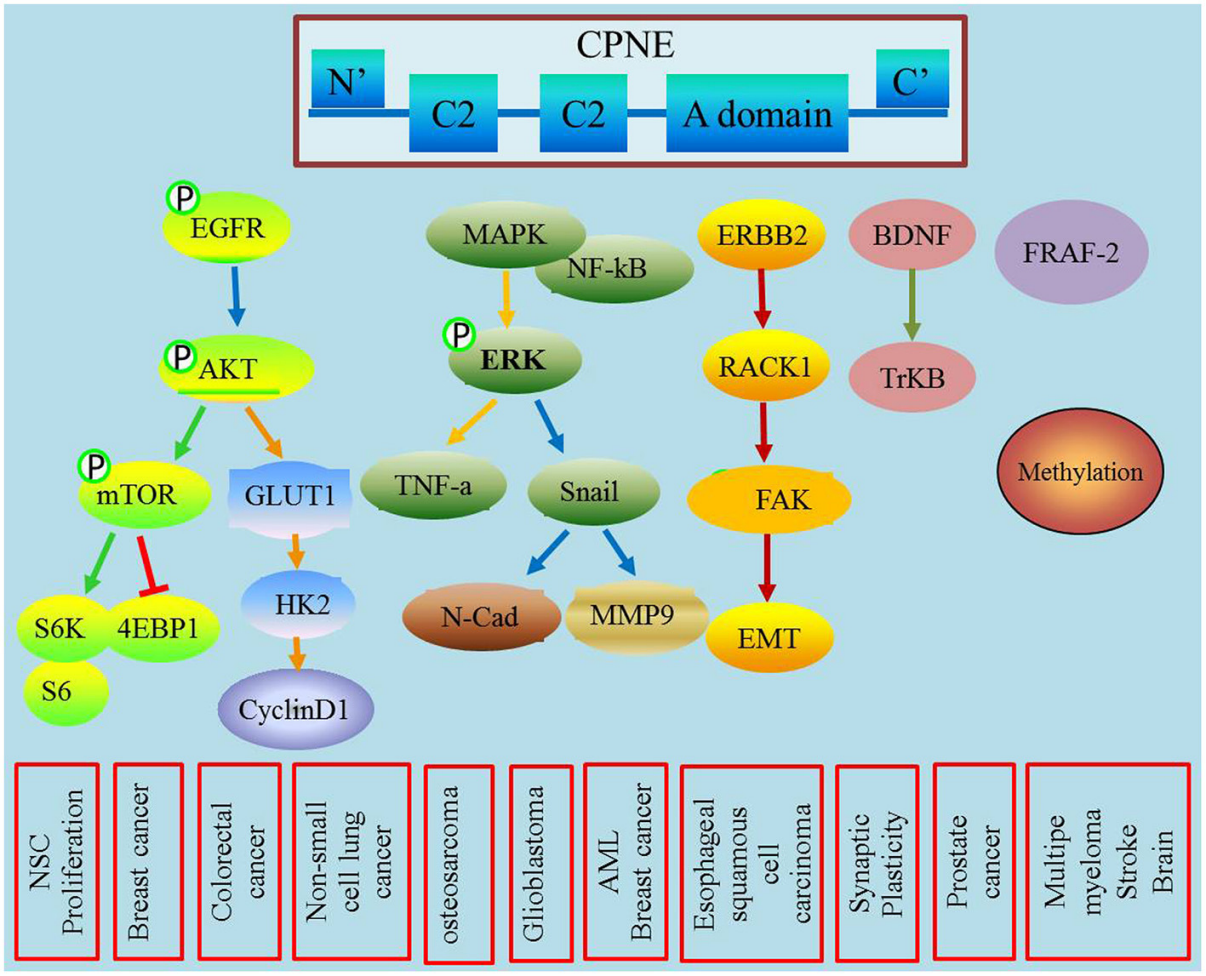
This figure describes the role of CPNEs in mediating tumor biological process and also illustrated the detailed involved signal transducer molecules.

CPNEs are a newly discovered class of phospholipid-binding proteins that are widely expressed, and their functions are Ca^2+^-dependent and structurally evolutionarily conserved (Creutz et al., 1998). The CPNEs consisted of nine family members (Tomsig et al., 2003). Although their exact functions and biological roles remain unclear, an increasing number of studies have shown that CPNEs may mediate a variety of signaling pathways involved in tumorigenesis and development (Figure 1). This review highlights the biological properties of the copine family and their roles in membrane trafficking, tumor progression and metastasis.

## The CPNE Family

The CPNE protein was originally discovered in nematodes and plants. Similar to other gene families, the CPNE family was also present throughout the evolution. CPNE1 was first reported in 1998, when Creutz isolated annexin in *Paramecium*. Nine CPNEs have been identified, and eight (CPNE1–8) are found in mammals (Tomsig and Creutz, 2000). CPNEs are highly homologous: CPNE2–5 exhibit 60%, 78%, 53% and 56% homology, respectively (Goel et al., 2019). The distribution of some CPNE genes is limited. Among them, CPNE1–3 are the most widely distributed, which expressed in almost all mammalian tissues, including brain, heart, lung, liver, intestine, spleen, testis and kidney. These genes induce the differentiation of granulocytes, and CPNE3 is expressed in the early stage of neutrophil differentiation (Cowland et al., 2003). CPNE4 is distributed in the heart and surrounding large blood vessels and cranial nerves (Goel et al., 2019). CPNE5 is mainly expressed in the lymphatic system, heart, stomach, spleen, lymph nodes and testis (Ding et al., 2008). CPNE6, which is also known as N-CPNE, is specifically expressed in brain neurons (Perestenko et al., 2010). CPNE6 has recently been found to be closely related to the learning and memory abilities of mice (Reinhard et al., 2016). In addition, CPNE6 expression is increased in epilepsy patients (Zhu et al., 2016). CPNE8 was initially identified as a major gene expressed in the prostate and testis. Further studies also found that CPNE8 is distributed in the prostate, heart and brain (Maitra et al., 2003).

## Molecular Structure and Biological Characteristics of CPNEs

CPNEs are mainly composed of the N-terminus and the C-terminus, and the C-terminus contains an A-domain, which is structurally related to the extracellular structure of integrin, and its main function is protein binding (Nalefski and Falke, 1996). The N-terminus contains two C2 domains that play a key role in regulating the binding of calcium and phospholipids (Rizo and Sudhof, 1998). These domains are present in an increasing number of membrane-bound proteins and involved in a variety of cellular signaling pathways and cellular processes, including membrane trafficking, lipid messenger production, GTPase activation and protein phosphorylation (Mukhopadhyay et al., 2017; Yanez Arteta et al., 2018). The C2 domains are characterized by an eight-chain antiparallel β-sandwich structure and are classified into two different topologies with slightly different positions and connectivity in the structure of the β-chain (Viaud et al., 2021). The C-terminus of CPNE shares similarity with the protein binding domain of certain integrin known as the A domain (Singh et al., 2017). The domains of human CPNE1, 2 and 4 mediate the binding of CPNEs to target proteins. More than 20 target proteins have been identified, many of which involve intracellular signaling pathways, such as MAPK, ERK, CDC42, and C12 ubiquitin (Gupta et al., 2010; Guo et al., 2020). In addition, CPNEs recruit these target proteins to the phospholipid surface, suggesting that they can modulate the activity and localization of these proteins in cells in response to changes in intracellular Ca^2+^ (Creutz et al., 1998). Based on the current findings, we propose a hypothesis that one function of CPNEs is to facilitate Ca^2+^-mediated regulation of intracellular signaling pathways (Ma et al., 2011). In addition, a variety of proteins have been identified as CPNE targets, many of which are involved in intracellular signaling pathways (Tomsig et al., 2004).

## The Role of CPNEs in Membrane Transport

CPNEs are involved in the regulation of membrane fusion mainly because the C2 domain binds to annexin on the membrane under conditions of Ca^2+^ stimulation, thereby affecting lysosomal and endosomal fusion and regulating autophagy (Ghislat and Knecht, 2012). CPNEs are soluble membrane proteins that contain two tandem C2 domains at the N-terminus and an A domain at the C-terminus (Damer et al., 2005). The C2 domain acts as a calcium-dependent phospholipid binding motif and may be involved in cellular signaling and membrane trafficking pathways (Park et al., 2014). The A domain, which is named after von Willebrand factor, is a plasma and extracellular matrix protein that has been studied in integrins and several extracellular matrix proteins and appears to play a role in protein binding (Whittaker and Hynes, 2002). CPNEs have multiple targets in cells and their expression can be regulated in response to the activation of signal transduction pathways through crosstalk mechanisms, and CPNEs may serve as a skeleton protein during the membrane transport process (Tomsig et al., 2004).

## The Role of CPNEs in Signal Transduction

Different CPNEs could have different roles in different signaling pathways, which are dependent on their calcium sensitivities, lipid specificities, and target proteins (Ilacqua et al., 2018). Tomsig JL and other studies reported that CPNE1 is involved in cell signal transduction processes. CPNE1 plays an important role in regulating neuronal differentiation of HiB5 cells, mainly by activating the AKT signaling pathway via interacting with JAB-1 and 14-4-3 gamma (Kim et al., 2018). CPNE1 regulates the NF-κB-associated proteins MAPK, ERK and other signaling molecules through the A domain, which leads to the activation of the downstream TNF-α signaling pathway (Ramsey et al., 2008). Recent *in vitro* studies of positional candidates confirm that CPNE1 and STC2 are regulators of myogenesis (Hernandez Cordero et al., 2019). CPNE3 promotes migration and invasion in non-small cell lung cancer by interacting with RACK1 via FAK signaling activation (Lin et al., 2018).

## The Role of CPNEs in Cancer

### CPNE1

In addition to its role in the central nervous system, accumulating studies have also highlighted its function in tumors. However, CPNE1 is rarely studied in tumors. Recent studies have shown higher CPNE1 expression in prostate cancer and that CPNE1expression is associated with the stage and prognosis of prostate cancer patients. Mechanistically, CPNE1 interacts with TRAF-2 to promote prostate cancer progression (Liang et al., 2017). In osteosarcoma, CPNE1 enhances cell proliferation and migration via the MAPK pathway and TGF-beta pathway (Jiang et al., 2018). Recent research revealed that CPNE1 promotes colorectal cancer progression by activating the AKT-GLUT1/HK2 cascade and enhances chemoresistance (Wang et al., 2021). Another study showed that CPNE1 promotes tumorigenesis and radioresistance in triple-negative breast cancer by regulating AKT activation, and targeted CPNE1 expression may be a strategy to sensitize triple-negative breast cancer cells to radiation therapy (Shao et al., 2020). For lung cancer, our previous studies demonstrated that CPNE1 is highly expressed in NSCLC tissues and is correlated with lymph node metastasis and poor survival in patients (Liu et al., 2018b). In addition, we also demonstrated that CPNE1 overexpression promotes cell proliferation and metastasis via the EGFR signaling pathway (Tang et al., 2018; Du et al., 2020). In hepatocellular carcinoma, overexpressed CPNE1 regulates the cell cycle process to mediate cell dedifferentiation (Skawran et al., 2008).

### CPNE3

Studies have confirmed that CPNE3 is associated with schizophrenia, but the specific mechanism remains unclear. Other studies have confirmed that CPNE3 participates in processes involved in acute myocardial infarction and coronary heart disease by potentially mediating the metabolism of nuclear fatty acids, serum total cholesterol and triglycerides (Tan et al., 2019). In addition, recent studies have also found that CPNE3 is highly expressed in breast cancer, prostate cancer and ovarian cancer and is involved in tumor cell proliferation and metastasis. Mechanistically, CPNE3 exhibits kinase activity, phosphorylates Hl histones and basic phospholipid proteins, activates downstream signaling pathways, and subsequently promotes tumor proliferation and metastasis (Thomas et al., 2008; Mo et al., 2013). Heinrich C found that CPNE3 induces EMT by activating the ErbB2 protein and induces tumor cell invasion and migration (Heinrich et al., 2010). Recent studies have shown that upregulated Copine3 with Jab1 activated downstream ErbB2 signaling and motility in breast cancer cell (Choi et al., 2016). Colorectal cancer patients with lower exosomal CPNE3 levels had substantially better disease-free survival and overall survival, implying that CPNE3 is a diagnostic and prognostic biomarker (Sun et al., 2019). In hepatocellular carcinoma, silencing the expression of CPNE3 enhances the sensitivity of cancer cells to the molecular targeted agent sorafenib (Chen et al., 2018). In NSCLC, CPNE3 expression level was positively correlated with clinical stage and TNM classification and quantitative proteomic analysis identifies CPNE3 as a novel metastasis-promoting gene in NSCLC (Lin et al., 2013). CPNE3 can promote migration and invasion in non-small cell lung cancer by interacting with RACK1 via FAK signaling activation (Lin et al., 2018). Upregulation of CPNE3 suppresses invasion, migration and proliferation of glioblastoma cells through FAK pathway inactivation (Shi et al., 2021). In addition, high expression of CPNE3 predicts adverse prognosis in acute myeloid leukemia (Fu et al., 2017).

### CPNE5

Umeda S showed that CPNE5 expression is decreased in esophageal squamous cell carcinoma (ESCC), suggesting shorter overall survival in patients. Multivariate analysis showed that low CPNE5 expression was an independent prognostic factor for OS. Moreover, low CPNE5 expression potentially promotes the local growth of esophageal cancer and increases resistance to chemotherapy drugs. These findings suggest that CPNE5 can be used as a biomarker for predicting ESCC recurrence, especially in patients with local recurrence, and can help ensure that patients receive optimal treatment and follow-up (Umeda et al., 2018). It is also reported that multiple myeloma (MM) patients with higher CPNE5 expressions had longer event-free survival and overall survival, suggesting that CPNE5 might be used as a positive indicator for MM (Yang et al., 2018).

### CPNE6

The report of CPNE6 in tumor progression in only limited in glioblastoma multiforme (GBM). GBM is a common type of brain tumor in adults; however effective candidate biomarkers for gene therapy in GBM remain unclear (Ni et al., 2018). It is reported that hub genes CPNE6, HAPLN2, CMTM3, NMI, CAPG, and PSMB8 were identified as potential liquid biopsy biomarkers for GBM diagnosis (Dent et al., 2008).

### CPNE7

CPNE7 is a candidate tumor suppressor gene in breast cancer tissue. CPNE7 exhibits high homology with other members of the copine family, such as CPNE1, CPNE3 and CPNE6. CPNE7 is considered a potential tumor suppressor gene (Savino et al., 1999). Sequencing analysis of bladder transitional cell carcinoma revealed 565 candidate gene mutations, including CPNE7 and serine/arginine repetitive matrix 5, suggesting that CPNE7 mutations may be related to important mechanisms involved in bladder cancer. Identification of these genes may have therapeutic significance and may contribute to the development of future treatments for bladder cancer (Pan et al., 2016). To date, the role of CPNE7 in promoting or inhibiting cancer has not been clear, and further research is expected to confirm this hypothesis.

### CPNE8

Ramsey found that in patients with acute myeloid leukemia (AML), the CPNE 8 gene can fuse with the AMLI gene to form an AML-CPNE8 chimera, thereby inhibiting AML gene transcription. It is hypothesized that CPNE8 negatively regulates the proliferation of AML cancer cells (Ramsey et al., 2003). A recent study showed that RP11-396F22.1 may represent an early diagnostic indicator of cervical cancer. After knocking down RP11-396F22.1, CPNE8 expression was significantly upregulated. It is hypothesized that CPNE8 is related to the occurrence of cervical cancer, but the specific mechanism requires further study (Zhao et al., 2018).

### CPNE9

Recent research found that high CPNE5 and CPNE9 expression might serve as positive indicators of multiple myeloma, and the expression of both genes was a better predictor of survival in multiple myeloma patients (Zhu et al., 2021). A study by Liu found that CPNE9 is specifically expressed in pancreatic tumor tissues, indicating that CPNE9 is related to cancer progression (Liu and Liu, 2015). Moreover, CPNE9 was among the top five genes of the prognostic 14-gene signature that was used to construct the prognostic model that demonstrated a high predictive ability for glioblastoma (Hou et al., 2019). This finding suggests that CPNE9 has important clinical significance for the prognostic assessment of glioblastoma patients.

## Prospects

Early diagnosis and precise treatment of tumors are directly related to patient prognosis. Therefore, early diagnosis of tumors and marker screening are particularly important. At present, no clear biomarkers are available for the diagnosis of lung cancer, and most of the existing studies are limited. The CPNE family is an important family that was discovered in recent years. CPNEs not only participate in the development and differentiation of the nervous system but also in the occurrence and development of numerous tumor types. CPNEs also affect the immune microenvironment of tumors. Studies on the roles of CPNEs in cancer are currently limited, and further research is needed. We hope that abnormal CPNEs expression will be validated as effective molecular markers for the early diagnosis, progression and prognosis of lung cancer.

## Author Contributions

HT: conception and design, manuscript writing, and review. PP and ZQ: data curation and analysis, study supervision. ZZ and QW: data curation and analysis. SS and FL: formal analysis, results discussion, and manuscript revision.

## Conflict of Interest

The authors declare that the research was conducted in the absence of any commercial or financial relationships that could be construed as a potential conflict of interest.
